# Time Series RNA-seq in Pigeonpea Revealed the Core Genes in Metabolic Pathways under Aluminum Stress

**DOI:** 10.3390/genes11040380

**Published:** 2020-04-01

**Authors:** Zhaoxu Gao, Biying Dong, Hongyan Cao, Hang He, Qing Yang, Dong Meng, Yujie Fu

**Affiliations:** 1 The College of Forestry, Beijing Forestry University, Beijing 100083, China; gaozx@pku.edu.cn (Z.G.); dongbiying1029@163.com (B.D.); hongyan89945@163.com (H.C.); mengdongjlf@163.com (D.M.); 2 State Key Laboratory of Protein and Plant Gene Research, School of Life Sciences, and School of Advanced Agricultural Sciences, Peking University, Beijing 100871, China; hehang@pku.edu.cn; 3 Peking-Tsinghua Center for Life Sciences, Academy for Advanced Interdisciplinary Studies, Peking University, Beijing 100871, China; 4 Beijing Advanced Innovation Center for tree Breeding by Molecular Design, Beijing Forestry University, Beijing 100083, China; 5 Key Laboratory of Forest Plant Ecology, Ministry of Education, Northeast Forestry University, Harbin 150040, China

**Keywords:** pigeonpea, aluminum (Al), time series, metabolic, stress

## Abstract

Pigeonpea is an important economic crop in the world and is mainly distributed in tropical and subtropical regions. In order to further expand the scope of planting, one of the problems that must be solved is the impact of soil acidity on plants in these areas. Based on our previous work, we constructed a time series RNA sequencing (RNA-seq) analysis under aluminum (Al) stress in pigeonpea. Through a comparison analysis, 11,425 genes were found to be differentially expressed among all the time points. After clustering these genes by their expression patterns, 12 clusters were generated. Many important functional pathways were identified by gene ontology (GO) analysis, such as biological regulation, localization, response to stimulus, metabolic process, detoxification, and so on. Further analysis showed that metabolic pathways played an important role in the response of Al stress. Thirteen out of the 23 selected genes related to flavonoids and phenols were downregulated in response to Al stress. In addition, we verified these key genes of flavonoid- and phenol-related metabolism pathways by qRT-PCR. Collectively, our findings not only revealed the regulation mechanism of pigeonpea under Al stress but also provided methodological support for further exploration of plant stress regulation mechanisms.

## 1. Introduction

Pigeonpea (*Cajanus cajan* L.) is a kind of economic crop that grows in the semiarid tropics. In India, it is the second most important food legume. Pigeonpea is a hardy plant with strong growth and good stress tolerance. It can be used as food, feed, fuel wood, green manure, and hedges. In addition, it also plays an important role in insect reproduction, soil and water conservation, and so on [1,2]. It is distributed in Asia, Eastern and Southern Africa, Latin America, and Caribbean countries, and its maturity has a large temporal variation [1,3]. To meet the growing demand for pigeonpea, many problems must be addressed in order to expand its cultivation area. Specifically, acidic soil is the biggest challenge for widespread cultivation [2].

Low phosphorus (P) levels and aluminum (Al) toxicity are two major adverse factors in acidic soil, which, together, restrict crop growth [4,5,6,7]. Aluminum toxicity is a serious problem that threatens crop productivity in acidic soil [2,8,9]. Many crops can be influenced by Al, and the growth of roots and shoots can be limited by it [9,10,11]. Aluminum toxicity is the key factor influencing wheat production in acidic soils [9]. Furthermore, vacuole-membrane-localized Sl-ALMT9 increases when there is an abundance of Al, thereby elevating Al resistance and determining fruit malate contents in tomato [12].

Many Al-related works in legumes have focused on gene regulation and transcriptional analysis. For Al stress, *CcSTOP1*, which is a C2H2-type transcription factor, regulates Al tolerance genes in pigeonpea [11]. Another study of Al toxicity in soybean has provided a new insight into the soybean MATE family regarding the response function to abiotic stresses. However, metabolic changes are very important under stress. Through the analysis of the absorption efficiency of nutrients under Al stress, genotypes with good tolerance to Al stress have been found. Tolerant genotypes (IPA 7-10, T7, 67 B, and GT 101E) accumulate significantly high amounts of nutrients (>1.5 times) compared with sensitive genotypes [2]. Except for the absorption of nutrients, metabolic changes are also vital processes under Al stress. The secondary metabolites produced in plants are important substances in the process of plant growth and development. Under the interactions between phosphorus and aluminum, the adenosine triphosphate (ATP) and adenylate energy charge (AEC) levels are significantly changed and have an effect on the anaplerotic metabolism of root zone CO_2_ [6].

Aluminum in soil not only affects plants in special areas, such as pigeonpea, but is also found in the growing environment of many crops, such as rice and tomato [6,12,13,14]. Additionally, *Arabidopsis*, as a model plant, has also provided a research basis for Al stress [15,16,17]. Al stress in *Arabidopsis*, rice, tomato, and pigeonpea has been found to cause aluminum-dependent root growth inhibition [2,6,17]. In addition, the transcription factors *STOP1* in *Arabidopsis* and *CcSTOP1* in pigeonpea have been proved to play an important role in regulating Al resistance [11,16]. The living environment of plants is a complex system. In addition to Al stress, there is also bacterial invasion. In rice and *Arabidopsis*, the regulation of resistance to bacterial and aluminum toxicity has been found to respond to Al and bacterial regulation at the same time [14,15].

The availability of high-throughput sequencing technologies [18] and the completion of a draft genome of pigeonpea [19,20,21] make it possible to rapidly detect transcriptional changes in pigeonpea. Differential gene expression analysis is often used to study developmental and stress-related regulations. Through comparative transcriptome analyses between pigeonpea (*Cajanus cajan* L.) and its wild relative *Cajanus platycarpus* (Benth.) Maesen under infection of *Helicoverpa armigera* (Hübner), many important genes have been identified [22]. The candidate genes for Fusarium wilt and sterility mosaic disease in pigeonpea have been identified by next-generation sequencing [23]. There are many similar works of time series sequencing for development and stress analysis in *Arabidopsis* [24,25]. Therefore, the transcriptome sequencing of time series is a good method to study the important pathways and functional changes under stress responses.

In the current study, we analyzed the whole transcriptome changes under aluminum response by time series RNA sequencing (RNA-seq) in pigeonpea. Three replicates ensured the credibility of the data. The correlation analysis of differentially expressed genes (DEGs) in time points for different responses showed that the differences between two time points were also obvious under Al stress. Further clustering analysis divided all the DEGs into 12 clusters. To mine the Al-response-related core genes, we performed functional analyses in independent clusters. The regulation of metabolic processes showed enrichment among all the related functions and had differences between clusters. Global pathway analysis coupled with differential expression characterization of the DEGs revealed some new factors that have profound effects on Al stress. In addition, we verified these key genes related to metabolism by qRT-PCR. Collectively, our results provide vital metabolic characteristics for the study of Al stress in pigeonpea and important research methods regarding time series data.

## 2. Materials and Methods

### 2.1. Plant Materials and Growth Conditions

In a sterile environment, pigeonpea seeds were first sterilized with 75% alcohol for 30 s, then sterilized with sodium hypochlorite for 6 min, and finally, the pigeonpea seeds were washed 5 times in sterile distilled water for 30 s each time. Pigeonpea seeds were then planted in sterile medium containing MS base salts, sucrose, and agar in a greenhouse. The temperature in the greenhouse was a constant 24 °C, the light conditions were light/dark cycles for 16 h/8 h, and the illumination was 5000 lux. When the pigeonpea seedlings were 3 weeks old, we selected pigeonpea seedlings with consistent growth status for Al treatment. According to our previous research, we performed Al treatment at a concentration of 50 μM [26]. Samples of roots were collected at 0, 3, 6, and 12 h after Al treatment, frozen immediately in liquid nitrogen, and then stored at −80 °C for RNA isolation.

### 2.2. High-Throughput RNA-Sequencing 

Three biological repeats were performed for four time points. Libraries were sequenced on a HiSeq 2000 system (Illumina) using the 150 bp Paired-End protocol. The outputs for 12 samples were about 84 G paired-end reads (2 × 150 bp). The reads were initially tested by fastQC (www.bioinformatics.babraham.ac.uk/projects/fastqc/). The adapter and low-quality reads (Q value < 20) were removed by Cutadapt [27]. In order to get higher-quality reads, we cut the first 9 bp from each read using our Perl script.

Then, the reads left were mapped to the *C.cajan*_V1.0 reference [21] using the Spliced Transcripts Alignment to a Reference (STAR) tool [28]. The parameters of STAR were the default, and the mapping rates were greater than 90%. After that, we used Cuffdiff [29] to calculate the significant DEGs. The screening criteria for the DEGs were fold change (FC) ≥ 2, Q value ≤ 0.05, and FPKM (Fragments Per Kilobase of exon model per Million mapped reads) greater than 1 in at least one sample.

### 2.3. Fundamental Analysis for RNA-seq Data

Principal Component Analysis (PCA) for the 12 samples was performed using the R package Psych. Consistency analysis between two samples was performed using the R packages Hmisc and Corrplot.

### 2.4. Cluster Analysis for DEGs

The cluster analysis was conducted using Mev [30]. The K-means method was selected to generate the clusters. The Violin and Heatmap plots were created using R.

### 2.5. Gene Function Analysis

The DEGs were annotated with a gene ontology (GO) number by script, and then the comparison was performed using WeGO [31]. The DEG terms were compared with a reference annotation as well as with each other. The GO enrichment plot was made using R.

### 2.6. Metabolic Pathway Analysis

We used Mapman [32] to do the pathway enrichment analysis. By blasting the protein of the pigeonpea genes with the *Arabidopsis* genes’ protein library, we found the sequencing similar genes. The evalue was less than 0.01. The max_target_seqs was set to 1. Then, the sequencing similar genes were used to map to the *Arabidopsis* annotation in Mapman. The pigeonpea genes were selected by enrichment results.

### 2.7. Plant RNA Extraction and qRT-PCR

Roots of pigeonpea were ground into powder in liquid nitrogen, total RNA was extracted from the samples using the CTAB method [33], while double-stranded cDNA was synthesized by the PrimeScript RT reagent kit with gDNA Eraser (Toyobo, Osaka, Japan). Then, NanoDrop 8000 (Thermo Fisher Scientific, Waltham, Massachusetts, USA) was used to evaluate the cDNA quality. The qRT-PCR experiments were performed using SuperReal PreMix (Probe) (Tiangen Biotech, Beijing, China) and CFX connect (Bio-Rad, West Berkeley, California, USA). The expression of actin was used as an internal standard. The data were analyzed using Bio-Rad software with the ddCT method. We used the Sangon Biotech website (http://www.sangon.com/newPrimerDesign) and primer 5.0 to synthesize the primers, and the primer information for qRT-PCR is listed in Table S3. All reactions were performed in triplicate. Data analysis was performed in EXCEL and GraphPad Prism 7 analysis software.

## 3. Results

### 3.1. Aluminum Treatment Aroused Genome-wide Changes in Pigeonpea

**Figure 1:**
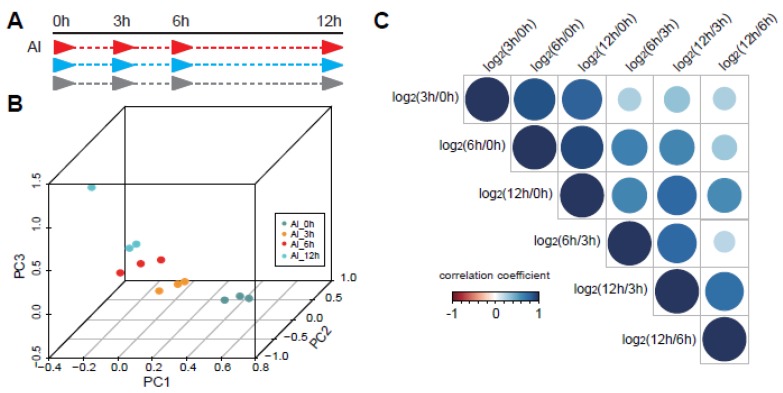
The time series sequencing showed dynamic regulation of Al response in pigeonpea. (**A**) The processing of Al response in pigeonpea. Three replicates were conducted for four time points, which were 0, 3, 6, and 12 h. (**B**) Three components of Principal Component Analysis (PCA) could divide the time series groups well. Different colors represent different processing time points. (**C**) The distribution of correlation coefficients between the differentially expressed gene (DEG) groups, which contain DEGs between 3 and 0 h, DEGs between 6 and 0 h, DEGs between 12 and 0 h, DEGs between 6 and 3 h, DEGs between 12 and 3 h, and DEGs between 12 and 6 h.

Under Al stress, the root growth of legumes is inhibited [10,11]. It can be saturated at a 20 μM concentration [10]. Al-inducible citrate secretion is an important change in pigeonpea. The initial time of citrate secretion change of pigeonpea under 100 μM Al stress is 3 h, and the trend of change can be maintained up to 12 h [10]. To further analyze the regulation mechanism under Al stress, we profiled the dynamic transcriptional analysis using time series RNA sequencing under Al responses. We surveyed the transcriptome changes at 0, 3, 6, and 12 h (Figure 1A). Three replicates were performed and the repeatability was good (Figure S1A). The raw data included ~21 M 150 bp paired-end reads in each sample. The meaning of the Q20 test is that approximately 99% of the reads have a high-quality score (greater than 20). The sequencing data passed the Q20 test and showed that the quality of the data was very good. After trimming the adapter and low-quality reads, the proportion of uniquely mapped reads was almost 90% by aligning to the *C.cajan*_V1.0 reference using STAR [28]. Then, there were 11,425 DEGs in at least one time point (Table S1). The three components of PCA could divide the time series groups well in comparison with the two-component PCA analysis (Figure 1B and Figure S1B).

In addition to the comparison between the treated samples and the control, we also made a comparative analysis among three Al processing samples, among which included 6062 DEGs at 3 and 0 h (group 1), 8073 DEGs at 6 and 0 h (group 2), 9231 DEGs at 12 and 0 h (group 3), 2368 DEGs at 6 and 3 h (group 4), 4782 DEGs at 12 and 3 h (group 5), and 1578 DEGs at 12 and 6 h (group 6) (Table S2). Through comparing the DEGs in the different groups mentioned above, we obtained the correlation coefficient among them (Figure 1C). The correlation values of DEGs between the first, second, and third groups were relatively large, which indicated that the DEGs at the later time point’s response to stress were relatively similar to the control sample (0 h), while groups 4–6 showed great divergence compared with other groups (Figure 1C). Those data suggested that the response to Al in pigeonpea showed significant dynamic patterns at different response time points. 

### 3.2. The DEGs Showed Dynamic Regulation of Stress

**Figure 2:**
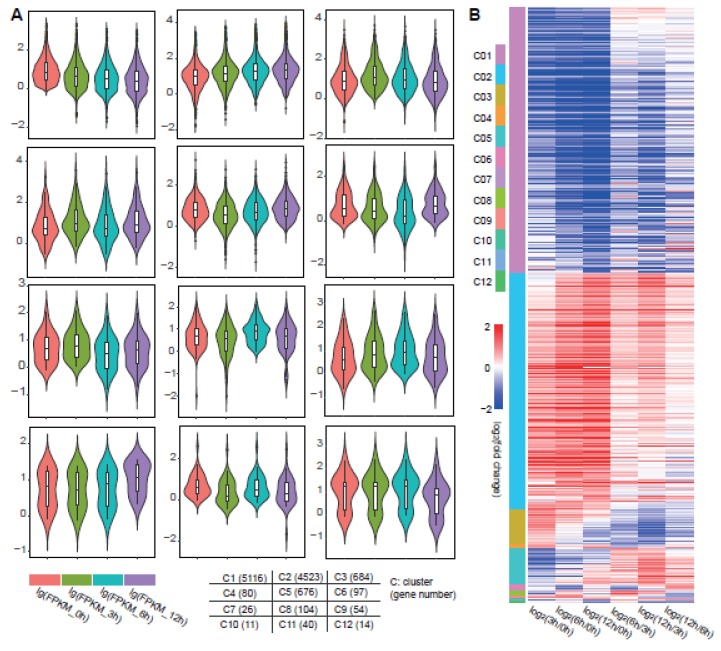
Total DEGs among the four time points were separated into 12 clusters by dynamic regulation pattern. (**A**) On the basis of FPKM for DEGs in each time point, there were 12 clusters in the time series response. The Violin diagram shows the expression level of genes in each group. The FPKM values were converted to lg forms to reduce the difference between outliers. Different colors represent four processing time points, which are 0, 3, 6, and 12 h from left to right. The tags and number of genes in each group are written in the grid graph at the bottom. (**B**) The differential expression pattern between two time points are shown in the Heatmap. The color column at the left shows the 12 clusters. The red–blue Heatmap contains the DEGs between 3 and 0 h, DEGs between 6 and 0 h, DEGs between 12 and 0 h, DEGs between 6 and 3 h, DEGs between 12 and 3 h, and DEGs between 12 and 6 h.

To further mine the core genes in the Al response, we separated those total DEGs into 12 clusters by their dynamic regulation patterns using Mev [30] (Figure 2A). The dynamic clusters were conducted by the absolute expression value FPKM for each gene at four time points. There were four clusters that had major genes (clusters 1–3 and 5), and the remaining clusters had minor genes. Simultaneously, the dynamic patterns in clusters 1 and 2 and clusters 3 and 5 were the opposite, respectively. Genes in cluster 1 continuously decreased in the response time points. Genes in cluster 2 steadily increased in the response time points. The genes in cluster 3 showed an increasing pattern in the early response time point and a decreasing pattern in the later response time points. The genes in cluster 5 showed a decreasing pattern in the early response time point and an increasing pattern in the later time points (Figure 2A).

According to the DEG cluster analysis, we analyzed the differential expression of genes in each cluster at different time points in a Heatmap pattern (Figure 2B). Similar to the previous overall comparison analysis results (Figure 1C), in the gene cluster analysis, the expression change pattern of the same gene in groups 1–3 was similar, but in the later response time points, the comparison between any two time points showed greater dynamic variation. Taken together, these observations revealed that the Al stress treatment had dynamic processing. There were some common stress signal reception and processing pathways, yet the specific genes that responded at three time points may have core roles in growth and development.

### 3.3. Dynamic Genes Triggered by Al treatment Correlate with Vital Functions

**Figure 3:**
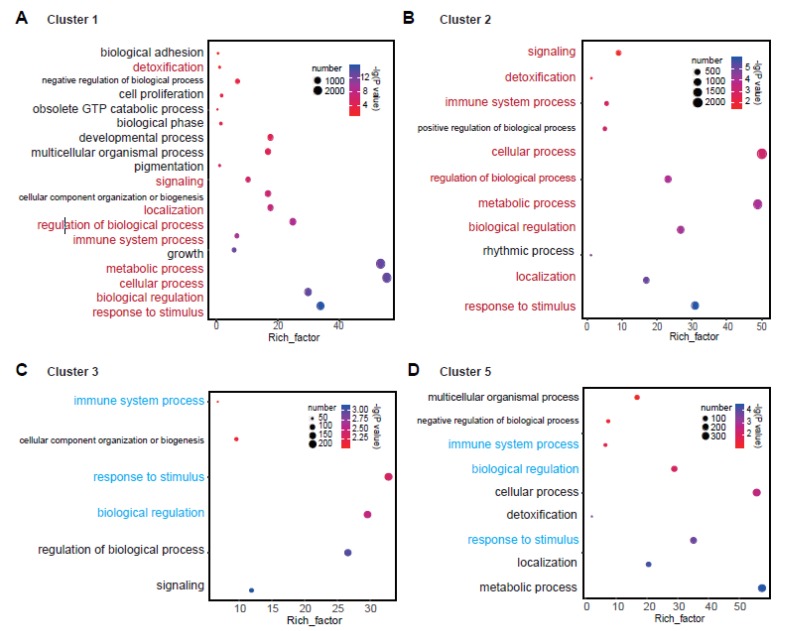
The corresponding biological function for clusters 1–3 and 5. (**A**) Gene ontology (GO) term for cluster 1. (**B**) GO term for cluster 2. (**C**) GO term for cluster 3. (**D**) GO term for cluster 5. The same GO terms between clusters 1 and 2 are marked in red. The same GO terms between clusters 3 and 5 are marked in cyan. The size of the circle represents the number of genes. The color of the circle represents the enriched *p*-value, which was converted to –lg (*p*-value). The *x* axis is the value of the Rich factor. The Rich factor is the proportion of enriched genes to DEGs.

A functional analysis was carried out for the DEGs. Because of the absence of pigeonpea species in the existing annotation database, we extracted the GO number of genes in each cluster and then compared them with the GO results of all genes in the pigeonpea reference to obtain the enriched gene functions in the clustered genes. The enrichment and significance test analyses were conducted in WeGO [31]. To get the distinct results from the GO function, we demonstrated the results by showing the enrichment number, proportion of enrichment, and the significance test *p*-value simultaneously (Figure 3). 

As previously mentioned, we compared the gene function between two opposite clusters (Figure 3). For clusters 1 and 2, nine GO terms were commonly enriched, such as response to stimulus, biological regulation, metabolic process, cellular process, immune system process, regulation of biological process, localization, signaling, and detoxification. In addition, there were some important enriched gene functions in cluster 1, such as growth, pigmentation, multicellular organismal process, developmental process, negative regulation of biological process, and so on (Figure 3A,B). 

For clusters 3 and 5, there were only three GO terms commonly enriched in the two clusters, which were immune system process, biological regulation, and response to stimulus. Further, some important functional terms existed in cluster 5, which were metabolic process, localization, detoxification, cellular process, negative regulation of biological process, and multicellular organismal process (Figure 3C,D). In the traditional way, the GO analysis was generally carried out by overall DEGs. Many detailed functions were embodied by the functional analysis of DEG groupings. We could see that it may find some specific enriched regulatory functions of gene sets, and it would be more accurate this way. These results confirmed that the functional enrichment of differentially expressed genes was also dynamic according to their different expression patterns. This result was very important for mining the key regulatory genes in the stress response process.

### 3.4. Metabolic Pathways were Significantly Enriched in the Core DEGs

**Figure 4:**
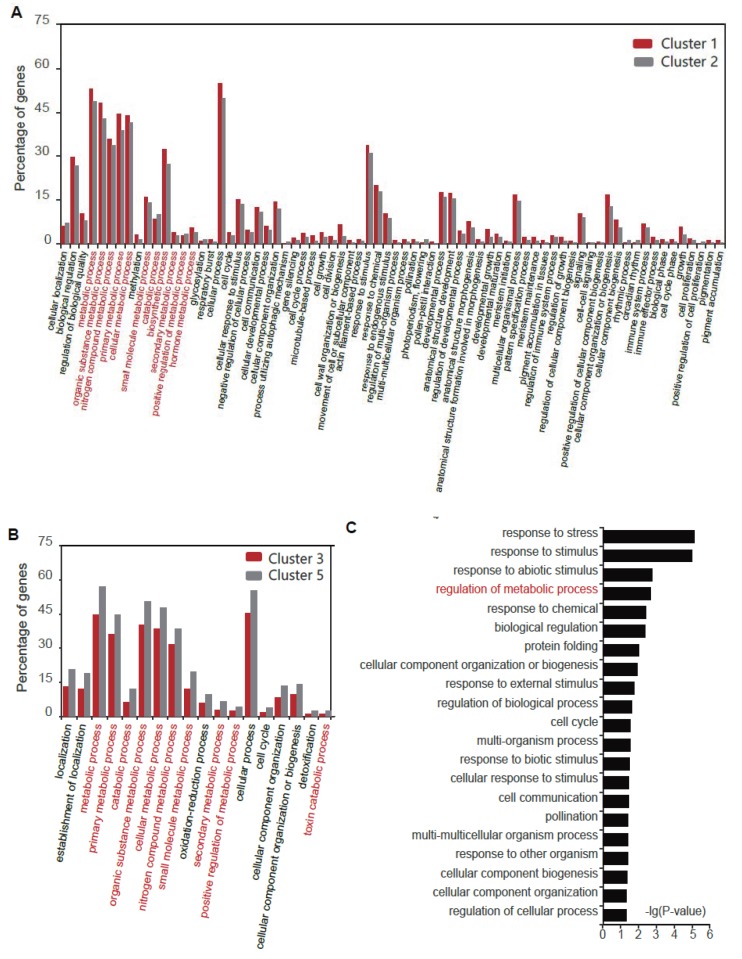
The comparison of GO for different clusters showed distinct functional differentiation. (**A**) GO term comparison for clusters 1 and 2. (**B**) GO term comparison for clusters 3 and 5. (**C**) GO term for genes in clusters except clusters 1–3 and 5. The GO terms related to metabolism in (**A**–**C**) are marked in red.

To make explicit the vital biological function changes in the Al response, we made a comparison analysis between clusters 1 and 2 and clusters 3 and 5, respectively. Except for the terms’ response to stimulus and development process, most of the differential functions were concentrated in metabolism-related functional terms (Figure 4). By counting the percentage of genes, the proportion value of genes in metabolism-related terms was relatively high for clusters 1 and 2 (Figure 4A). Further, the main difference between clusters 3 and 5 was also the metabolism-related terms (Figure 4B). 

However, there were also other gene clusters with diverse expression patterns. Further on, we performed GO term analysis for genes in clusters except clusters 1–3 and 5. We found a minor proportion of terms related to the metabolic process (Figure 4C). Thus, the metabolism-related terms were the main changes under Al stress. 

**Figure 5:**
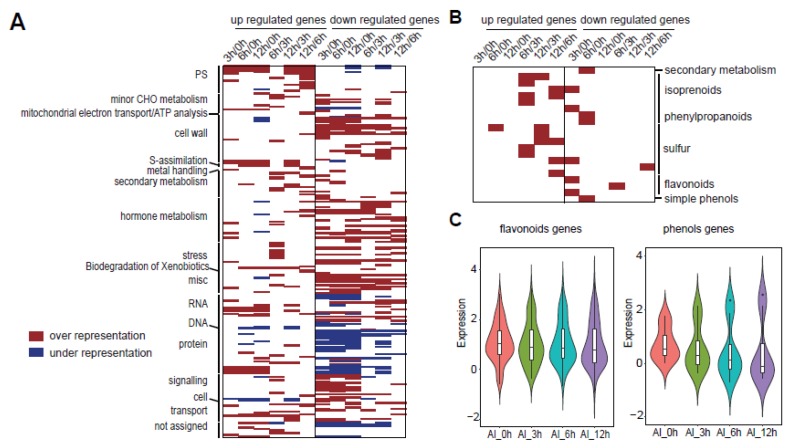
Key cluster (clusters 1–3 and 5) gene enriched pathways found by Mapman. (**A**) The total enriched pathways for these key cluster genes. Datapoints in each bin which exceeded the value of 1.0 were tested for over- or under-representation using Fisher’s exact test. Red represents the over-representation of genes in the corresponding pathways compared with the reference annotation. Blue represents the under-representation of genes in the corresponding pathways compared with the reference annotation. The genes were separated into two parts, which were upregulated and downregulated genes. (**B**) The magnification of metabolic pathways containing important flavonoid and simple phenol pathways. (**C**) The expression pattern of flavonoid-related genes and phenol-related genes extracted from (**B**). The *y* axis is for the lg(FPKM) in each time point.

To further analyze the roles of the metabolic process in Al stress, we mapped these metabolism-related genes to the metabolic pathways using Mapman [32]. Mapman is plant-specific software that can quickly annotate genes into various regulatory pathways, and it supported the visualization of gene differential expression data. Mapman provided a complete classification of gene function and a comprehensive picture of the pathways. Due to the lack of pigeonpea annotation information in Mapman, we obtained *Arabidopsis* genes similar to those of pigeonpea by Blast comparison. Then, we obtained the final lists for each DEG group with the *Arabidopsis* genes’ ID and corresponding pigeonpea genes’ expression. Based on the results in the above analysis, metabolism-related genes mainly existed in clusters 1–3 and 5, so we mainly analyzed these pathways with genes in the above four clusters. In the overall results, we divided the genes into upregulated and downregulated parts to see the enrichment levels in these pathways. At the same time, in enrichment analysis, the under-represented part was also identified; that is, the enrichment of this part of the genes was decreasing compared with the reference annotation (Figure 5A and Figure S2A). Among the pathways related to metabolism, we were interested in two pathways, which were flavonoids and phenols (Figure 5B and Figure S2B). To gain further insight into these two meaningful pathways in Al stress, we extracted those genes from the enriched pathways. Heatmap analysis showed an overall decreasing tendency in Al stress (Figure 5C). These results demonstrated that a certain number of vital genes in the metabolic pathway had a downward trend under the condition of Al stress.

### 3.5. The Selection of Core Genes Related to Metabolism

**Figure 6:**
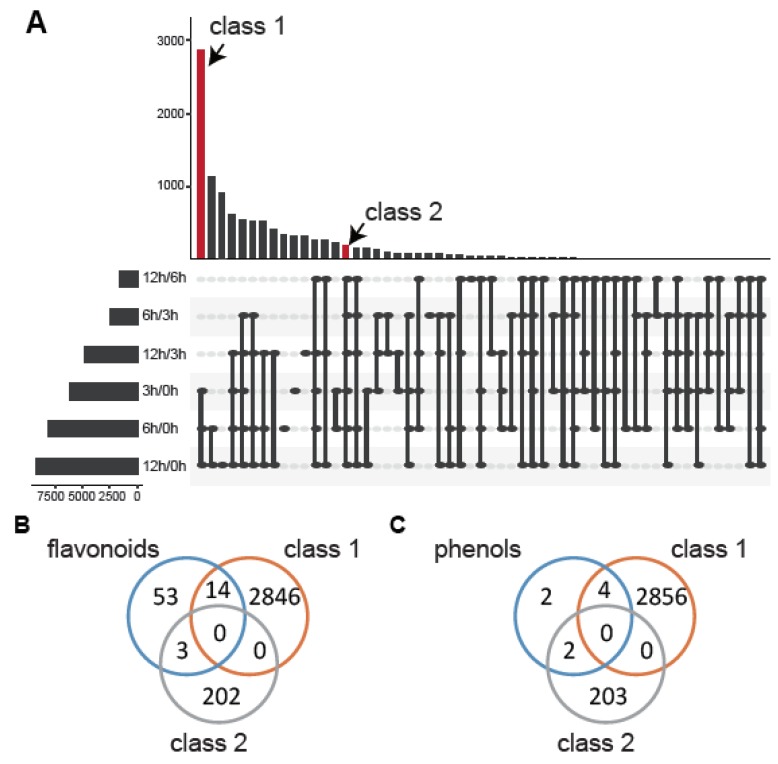
Selection of metabolic genes under Al stress. (**A**) The Venn analysis for the DEGs of different groups (3 h/0 h, 6 h/0 h, 12 h/0 h, 6 h/3 h, 12 h/3 h, and 12 h/6 h). Class 1 is the DEGs that appeared in the groups 3 h/0 h, 6 h/0 h, and 12 h/0 h. (**B**) The core genes in the flavonoid-related pathway. (**C**) The core genes in the phenol-related pathway.

To mine the mechanism between the metabolic pathway and Al stress, we performed a deep analysis of the differential expression of these genes. First, we classified the DEGs that differentially expressed at two time points (Figure 6A). Fifty-five classes were divided by Venn analysis, among which the common DEGs in 3 h/0 h, 6 h/0 h, and 12 h/0 h were the most enriched (class 1). The genes that appeared in all the differential expression groups (class 2) were more robust than others under Al stress. In the results, we selected the genes that were differentially expressed in 12 h/0 h, 6 h/0 h, 3 h/0 h, 12 h/6 h, 12 h/3 h, and 6 h/3 h at the same time or the genes that were differentially expressed in 12 h/0 h, 6 h/0 h, 3 h/0 h at the same time as the basic candidate genes. Then, we compared the flavonoid-related genes and phenol-related genes with these two classes, respectively. After that, fewer core genes were left, which were LOC109816497, LOC109807857, LOC109817906, LOC109792762, LOC109793867, LOC109816504, LOC109798137, LOC109802374, LOC109799422, LOC109817632, LOC109816540, LOC109803177, LOC109798064, LOC109817877, LOC109788985, LOC109813197, and LOC109797501 for flavonoids (Figure 6B), and LOC109807437, LOC109798912, LOC109816288, LOC109814583, LOC109816049, and LOC109816051 for phenols (Figure 6C). 

### 3.6. The Validation of Core Genes Related to Metabolism under Al Stress

**Figure 7:**
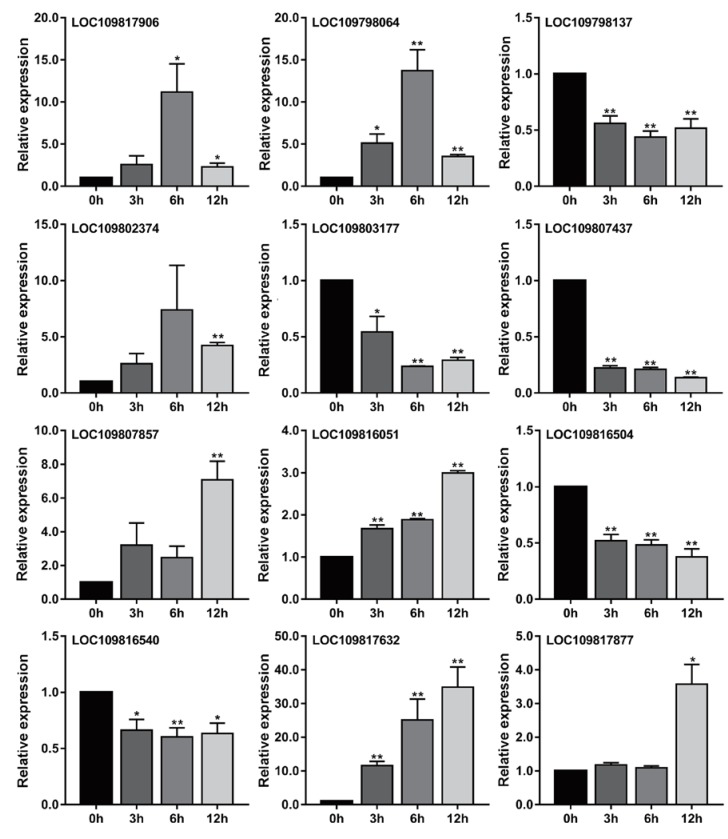
Relative expression levels of 12 core genes related to flavonoids and phenols in response to Al stress were determined by qRT-PCR. The levels of transcription of these genes in the roots of pigeonpea were studied at 0, 3, 6, and 12 h under Al stress. Each group had three biological replicates. **, *p* values < 0.01, and *, *p* values < 0.05, indicating significant difference from corresponding controls, *t*-test of independence.

We found that among these core genes related to flavonoids and phenols, many genes belonged to the same gene family. These core genes belonged to nine gene families, which were the 2-oxoglutarate (2OG) and Fe(II)-dependent oxygenase superfamily, the chalcone and stilbene synthase family, the HXXXD-type acyl-transferase family, the laccase gene family, the NAD(P)-binding Rossmann-fold superfamily, the oxidoreductase family acting on NADH or NADPH, the senescence-related gene family, the flavonoid 3-o-glucosyltransferase family, and the UDP-glycosyltransferase superfamily. In order to further verify the relationship between these genes and metabolism, we selected two genes from each gene family separately based on transcriptome data: a positive regulatory gene with the highest expression level at 12 h and a negative regulatory gene with the highest expression levels at 0 h. According to this principle, we screened 12 genes, and then we used qRT-PCR to detect the relative expression of these genes under Al stress. The results are shown in Figure 7. The relative expression trends of all genes at 3, 6, and 12 h were roughly consistent with the transcriptome data (Figure 7 and Table S1).

Among these nine gene families, all gene families except the laccase family were related to flavonoids. Among them, the 2OG and Fe(II)-dependent oxygenase superfamily, the chalcone and stilbene synthase family, the HXXXD-type acyl-transferase family, the flavonoid 3-O-glucosyltransferase family, and the UDP-glycosyltransferase superfamily had been reported and they were related to flavonoids. In plants, the 2OG and Fe(II)-dependent oxygenase superfamily plays an important role in many biosynthetic pathways, including biosynthetic pathways that lead to collagen, β-lactam antibiotics, and many modified amino acids and peptides [34]. Some iron 2-oxoglutarate-dependent oxygenases play an important role in catalyzing the penultimate step in anthocyanin flavonoid biosynthesis [35], and some chalcone and stilbene synthases can convert phenylpropane-CoA esters to flavonoids [36]. The HXXXD-type acyl-transferase family, the flavonoid 3-O-glucosyltransferase family, and the UDP-glycosyltransferase superfamily could modify the acylation and glycosylation of secondary metabolites such as lignin, flavonoids, and alkaloids [37,38,39]. The effects of these gene families have been reported in the synthesis and modification of the anthocyanin class of flavonoids, suggesting that the content of the anthocyanin class of flavonoids in pigeonpea may be changed after Al stress. 

In addition, we found that all core genes related to phenolics under aluminum stress belonged to the laccase family. Regarding the effect of laccase on phenols, it is generally believed that laccase participates in the anabolic metabolism of phenolic polymers, leading to the morphogenesis of plant fibers. Detailed analysis of phenolic compounds in plant tissues clearly showed that laccase mediated the polymerization of low-molecular-weight natural phenols in apoplastic bodies. In addition, laccase purified from plant tissues can use monolignol as a substrate and subsequently produce a coupling reaction to form lignin, but whether laccase in plants can convert monolignol to lignin has not yet been clearly reported [40,41,42,43].

In summary, the core genes related to flavonoids and phenols that we have screened provide some clues for analyzing the mechanism of flavonoids and phenols in aluminum stress in pigeonpea. All the analysis methods in this study present a useful reference for exploring the relationship between plant secondary metabolites and stress resistance.

## 4. Discussion

### 4.1. Dynamic Changes Can Increase the Plasticity under Al Stress

For the analysis of stress response, the time series sequencing method has been used in many works to study changes at the transcriptional level [24,25,44,45]. Most of these studies compared processed samples with a control sample to obtain the differentially expressed genes. However, gene changes are continuous under the process of stress response, so comparing the expression changes among the processing time points of stress could provide more dynamic change information. In our work, we found that the most dynamic changes appeared in the transitions of 3–6 h and 6–12 h (Figure 1C). Other comparisons between 3 and 0 h, 6 and 0 h, and 12 and 0 h were similar (Figure 1C). When we grouped the differentially expressed genes, we could see the dynamic changes of these genes more intuitively (Figure 2). In different stages of growth and development, the same gene will produce plasticity with changes of the environment. This method of continuous sampling at different time points could reveal the key links in the development regulation process [24]. In our results, we found that the plasticity brought by dynamic regulation of genes was also very important at different stages of stimulus response (Figure 1 and Figure 2). Thus, many important regulatory pathways were contained in these dynamic differentially expressed genes (Figure 3).

### 4.2. Metabolic Pathways Play an Important Role in Al Stress

Under biotic and abiotic stresses, plants have developed complicated signaling pathways to adapt. Currently, most research on this subject focuses on gene families in the signaling pathways [10,46,47,48] or gene transcriptional and post-transcriptional regulations [47]. Al stress had an important effect on the roots of legumes and the production of response substances. We sequenced and analyzed the global transcriptome with time series data under Al stress in pigeonpea (Figure 1). As mentioned above, we found many important regulatory pathways in our dynamically changing DEGs, for example, biological regulation, localization, response to stimulus, metabolic process, and detoxification (Figure 3). 

Through the functional comparative analysis of important grouping genes, we found that there were significant differences in important metabolism-related pathways (Figure 4). This result corresponded to the previous findings that Al stress affected the absorption of nutrients by roots [2,6]. Metabolic processes are essential for plant growth and development, so we further explored vital genes in these pathways (Figure 5 and Figure 6). Our results revealed that these vital genes in the metabolic pathways may have important regulatory roles under Al stress. 

### 4.3. Decreased Pattern of Important Response Genes in Metabolic Pathways is a Protective Mechanism in Plants

A certain number of the important genes related to metabolism found in our study were downregulated (Figure 2 and Figure 5), which was related to the negative effect of thes Al condition on the growth and development of pigeonpea [4,5,6,7]. Unlike animals, plants cannot move when the living environment changes. Some common changes in animals and plants include transcriptional regulation of transcription factors [10], alternative splicing [47], and miRNA regulation [25,44], while some studies have focused on important developmental pathways. Mitogen-activated protein kinase kinase kinase (MAPKKK) family members were found to be related to drought tolerance in maize through RNA-seq analysis [48]. In our results, we found gene disturbance in flavonoid- and phenol-related metabolism pathways. Flavonoids and phenols are important for metabolism in pigeonpea. Under stress, plants may reduce more secondary products to reserve energy.

In summary, we found that genes that had dynamic changes among the response time points under Al stress in pigeonpea. Further functional analysis showed that many pathways contribute to the stress responses. The metabolism-related pathways were the most significant differential pathways between DEGs. The core genes in metabolism-related pathways, containing flavonoids and phenols, were verified to demonstrate a vital role under Al stress. This finding sheds new light on Al stress in pigeonpea and other plants.

## Supplementary Materials

The following are available online at https://www.mdpi.com/2073-4425/11/4/380/s1, Table S1: Total DEGs; Table S2: Time series DEGs; Table S3: Primer sequence list for qRT–PCR analyses; Supplemental Figure S1: The foundational analysis of the data; Supplemental Figure S2: Mapman can map the metabolism related genes in the related pathways.
