# Supplementary material for: Time Series RNA-seq in Pigeonpea Revealed the Core Genes in Metabolic Pathways under Aluminum Stress
Source: Genes (Basel). 2020 Apr 1;11(4):380. doi: 10.3390/genes11040380 (PMC7230159; doi:10.3390/genes11040380)
Supplement: Supplementary file 1 [file genes-11-00380-s001.zip › genes-722755-supplementary/sup/figures.pdf]

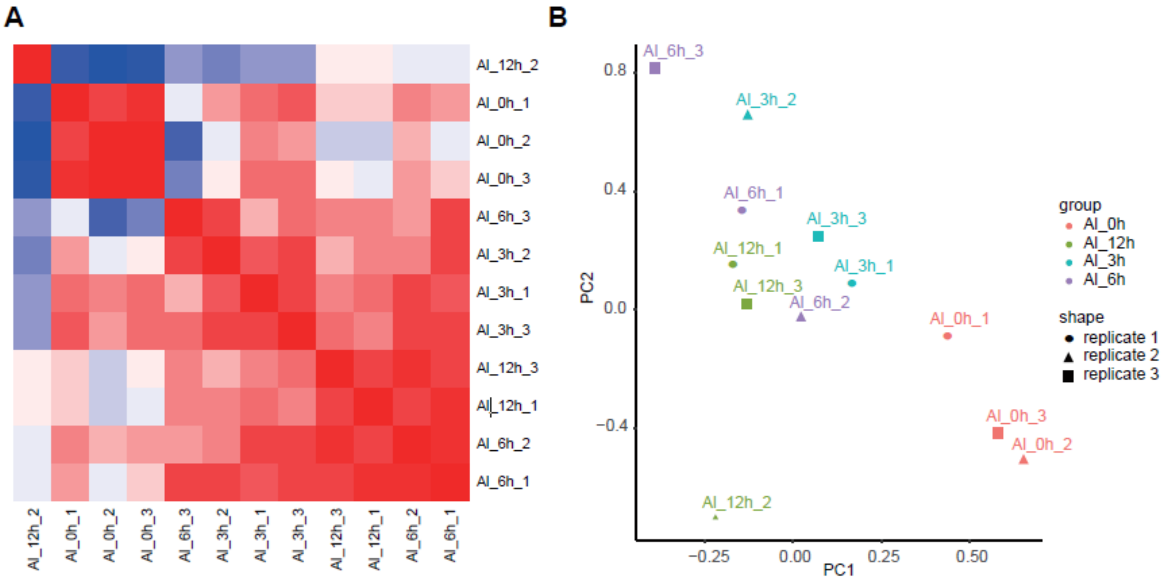


**Supplemental figure 1. The foundational analysis of the data.** (A) The repeatability of the three repeats of samples. Samples in 0h and 3h could cluster well. One replicate in 6h and 12h had one outline, respectively. (B) Two component analysis could not completely separate samples with different hourly responses. Different colors present different response time points. Different shapes present three replicates.

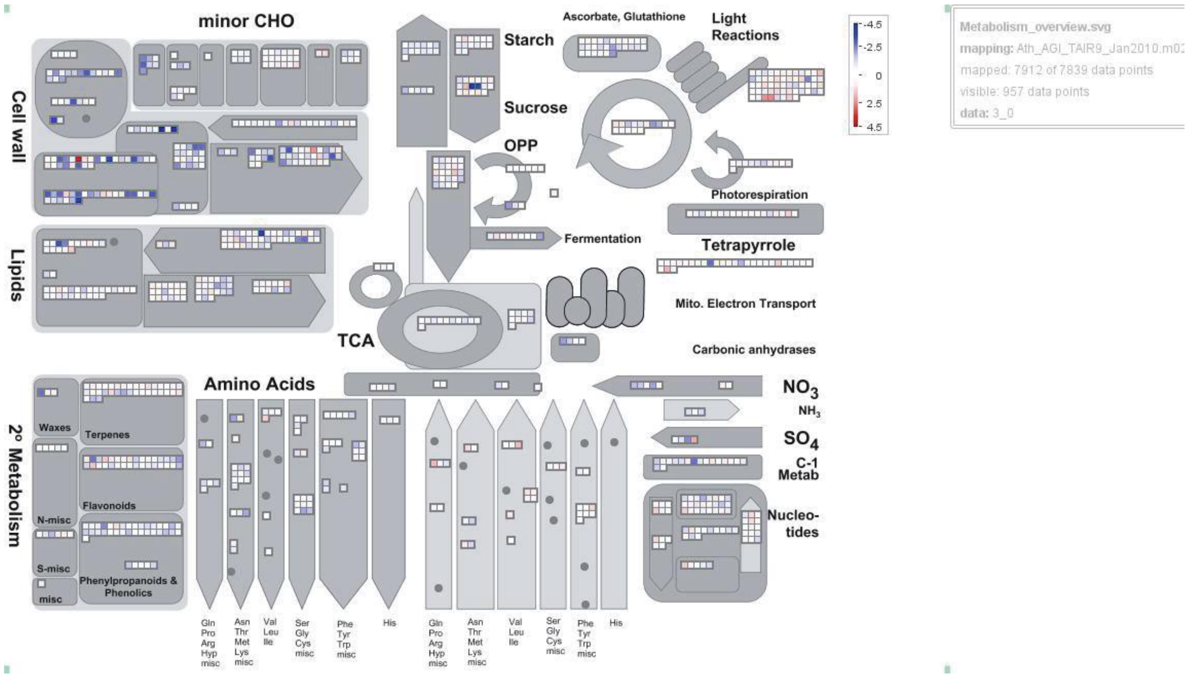

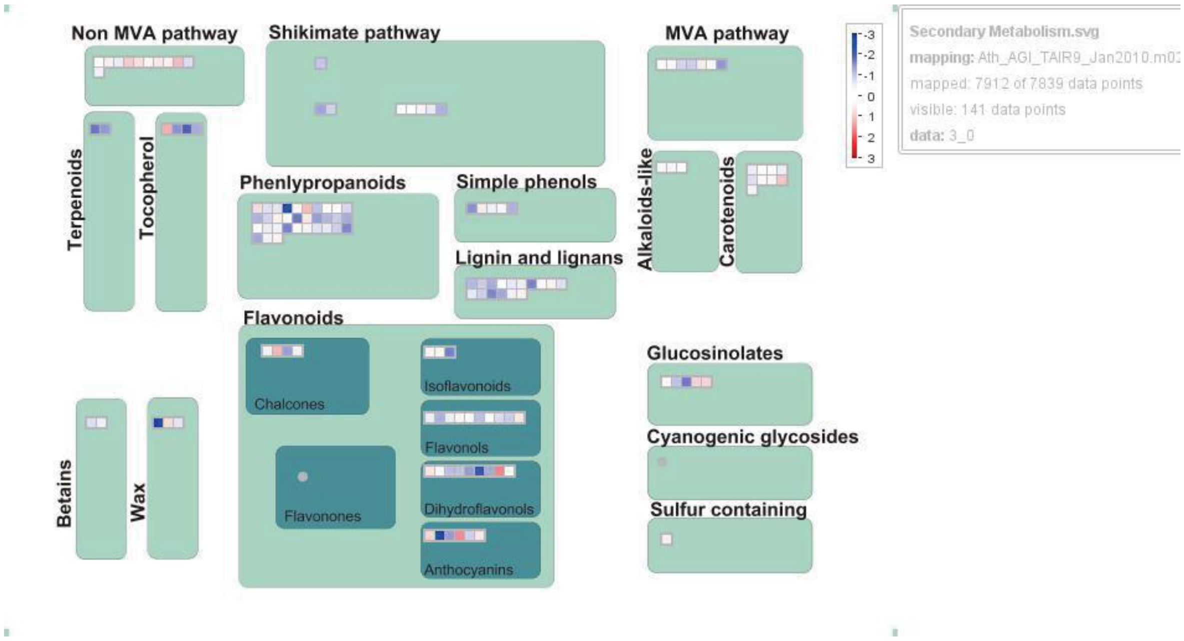


**Supplemental figure 2. Mapman can map the metabolism related genes in the related pathways. A.** Metabolism pathway. **B.** Secondary metabolism pathway. The genes present dynamic expression in 3h/0h. Blue was for down regulated. Red was for up regulated.
