# Supplementary material for: Time Series RNA-seq in Pigeonpea Revealed the Core Genes in Metabolic Pathways under Aluminum Stress
Source: Genes (Basel). 2020 Apr 1;11(4):380. doi: 10.3390/genes11040380 (PMC7230159; doi:10.3390/genes11040380)
Supplement: Supplementary file 1 [file genes-11-00380-s001.zip › genes-722755-supplementary/sup/Table S3. Primer.docx]

Table S3. Primer sequences list for qRT–PCR analyses

| Gene name | Primer-F（5'-3'） | | Primer-R（5'-3'） | |
| --- | --- | --- | --- | --- |
| Actin | CATGCCATCCTCCGTCTTGACTTAG | GCTCGGCTGTGGTGGTGAATG | |  |
| LOC109798064 | CCACCACGCCAACAGATACAGAG | CGGATTCCATTCGGACCAACGG | |  |
| LOC109798137 | TGCCTGGTGCGGATTATCAACTTG | TCCAGCATTGTTCTCAGCAAGGTC | |  |
| LOC109802374 | GCCACGCAACAAGCAGCAATG | GAGGACGAAATGGTGGTCGATGAG | |  |
| LOC109803177 | AGCCTTGTTACTGCCGAAAGACC | CATTGCTTCTCTTGTGGCTTGCG | |  |
| LOC109807437 | AAGCCTTTCACCACCAATGTCCTC | CACGCGCCGCCATGTAGTATC | |  |
| LOC109807857 | AGAAGATGAGAGCCACCAGACAGG | AAAGAGCACACCCCAATCAAGACC | |  |
| LOC109816051 | CGCACTCGTAACCACAAACCAAAC | GGCATGATGGGTGACGCTGAC | |  |
| LOC109816504 | CTTCTATCACCGCCTCAACACTGG | AGGCTTCCCAAACTGCTCTTCAAG | |  |
| LOC109816540 | CACAGCATCTTTGCCACAAGTTCC | GCACGCATGATCCAGCTTCTCTAG | |  |
| LOC109817632 | ACCACTTCCTCCAAACAGCACTTC | GGTTGCGGCGGAACGATGAG | |  |
| LOC109817877 | GCATCCAAAGCACAAGCAGAGAAG | AGAAGGGCCAGTTGTGAGAGTAGG | |  |
| LOC109817906 | GTGGTTCGGGAATTGGGGTTGAG | TGGCTCTGGGCATGGTGGATAG | |  |
